# Variability of antibiotic prescribing in patients with chronic obstructive pulmonary disease exacerbations: a cohort study

**DOI:** 10.1186/1471-2466-13-32

**Published:** 2013-05-31

**Authors:** Rachael Boggon, Richard Hubbard, Liam Smeeth, Martin Gulliford, Jackie Cassell, Susan Eaton, Munir Pirmohamed, Tjeerd-Pieter van Staa

**Affiliations:** 1 Clinical Practice Research Datalink, Medicines and Healthcare products Regulatory Agency, 151 Buckingham Palace Road, London SW1W 9SZ, UK; 2 Utrecht Institute for Pharmaceutical Sciences, Utrecht University, Utrecht, the Netherlands; 3 Division of Epidemiology and Public Health, Nottingham University, Nottingham, UK; 4 London School of Hygiene & Tropical Medicine, London, UK; 5 Primary Care and Public Health Sciences, King’s College, London, UK; 6 Division of Primary Care and Public Health, Brighton and Sussex Medical School, University of Brighton, Brighton, UK; 7 The Wolfson Centre for Personalised Medicine, Institute of Translational Medicine, University of Liverpool, Liverpool, UK

**Keywords:** Chronic obstructive pulmonary disease, Disease exacerbation, Clinical practice variation, Anti-bacterial agents, Primary health care, General practice

## Abstract

**Background:**

The role of antibiotics in treating mild or moderate exacerbations in patients with acute chronic obstructive pulmonary disease (COPD) is unclear. The aims were to: (i) describe patient characteristics associated with acute exacerbations amongst a representative COPD population, (ii) explore the relationship between COPD severity and outcomes amongst patients with exacerbations, and (iii) quantify variability by general practice in prescribing of antibiotics for COPD exacerbations.

**Method:**

A cohort of 62,747 patients with COPD was identified from primary care general practices (GP) in England, and linked to hospital admission and death certificate data. Exacerbation cases were matched to three controls and characteristics compared using conditional logistic regression. Outcomes were compared using incidence rates and Cox regression, stratified by disease severity. Variability of prescribing at the GP level was evaluated graphically and by using multilevel models.

**Results:**

COPD severity was found to be associated with exacerbation and subsequent mortality (very severe vs. mild, odds ratio for exacerbation 2.12 [95%CI 19.5–2.32]), hazard ratio for mortality 2.14 [95%CI 1.59–2.88]). Whilst 61% of exacerbation cases were prescribed antibiotics, this proportion varied considerably between GP practices (interquartile range, 48–73%). This variation is greater than can be explained by patient characteristics alone.

**Conclusions:**

There is significant variability between GP practices in the prescribing of antibiotics to COPD patients experiencing exacerbations. Combined with a lack of evidence on the effects of treatment, this supports the need and opportunity for a large scale pragmatic randomised trial of the prescribing of antibiotics for COPD patients with exacerbations, in order to clarify their effectiveness and long term outcomes whilst ensuring the representativeness of subjects.

## Background

Chronic obstructive pulmonary disease (COPD) is a progressive condition characterised by an obstructive pattern of expiratory airflow limitation which can’t be fully reversed. An estimated 3 million people have COPD in the UK with associated annual healthcare costs of £800 million [1,2]. COPD patients frequently suffer acute exacerbations of the disease, characterised by an increase in dyspnoea, sputum volume or purulence. Exacerbations are often bacterial in origin and antibiotic therapy is appropriate [3]. However, exacerbations may also be due to viral infections of the upper respiratory tract or may be non-infective. The role of antibiotics in treating mild or moderate acute exacerbation is unclear [4]. Most randomised trials evaluating antibiotics for COPD were restricted to patients hospitalised for acute exacerbations [5]. Of the 11 studies included in a recent review only 2 included patients from a GP setting, despite much healthcare for COPD patients being administered in this environment. Also, few of the studies provided details on resource utilisation associated with exacerbations or the effect of prescribing antibiotics on this utilisation [5].

Databases of electronic health records (EHR), such as those managed by the Clinical Practice Research Datalink (CPRD), provide the opportunity to conduct a large pragmatic randomised trial evaluating effects of antibiotics in patients with mild or moderate acute COPD exacerbations [6]. Participants can be recruited at the point of care and followed using routinely collected EHR. A key requirement for pragmatic trials is that ‘usual conditions’ apply to the research setting [7,8]. The selection and monitoring of patients should ideally mimic the ‘real world’. The only difference should be the random allocation of the interventions rather than the investigators deciding how to treat patients. Such a trial is currently being conducted within CPRD, and the present study was initiated in order to provide background information about current medical practice and outcomes of COPD exacerbations. The objectives were to: (i) describe the patient characteristics associated with acute exacerbations amongst a representative COPD population, (ii) explore the relationship between COPD severity and outcomes amongst patients with exacerbations, and (iii) quantify variability by practice in prescribing of antibiotics for exacerbations.

## Methods

This study used data from the UK General Practice Research Database (GPRD), the foundation of the CPRD primary care data. The database comprises computerised medical records maintained by GPs. The data, prospectively recorded since 1987, include demographics, prescriptions, clinical events, preventive care, specialist referrals, hospital admissions and major outcomes and covers 8% of the UK population [9]. A recent review of validation studies found that CPRD medical data were generally high quality [10].

CPRD primary care patients have been linked to hospital admission (Hospital Episode Statistics [HES]) and death certificate data. The linkages are performed using patients NHS numbers, dates of birth, sex and postcodes. HES collect dates of admission and discharge and main diagnoses, extracted from the medical records by coding staff. The death certificates list the date and causes of death. Linked data were available for 48% of practices (309 of the 639 practices; only practices in England willing to provide patient identifiers to the Trusted Third Party were included), shown to be representative of practices as a whole [11]. The study protocol was approved by the CPRD Independent Scientific Advisory Committee. All data was managed and analysed using Stata version 11.2 (Copyright 2009 StataCorp LP, Texas, USA).

### Cohort 1: COPD population

The study population included patients aged ≥40 years with a history of COPD in the clinical or referral files of the primary care data (as defined by the Quality and Outcomes Framework diagnostic Read codes [12]) who were eligible for linkage. The population was restricted to patients with at least nine months of active registration during 2005 to 2010, defined as the period between (a) the maximum of the patient’s current registration date and the practice “up-to-standard” date, and (b) the minimum of the date at which the patient left the practice and the last CPRD collection date from that practice. A random date was selected within the active registration period for each patient, ensuring at least six months of prior registration, and three months of follow-up during 2005 to 2010. This was done by first calculating the available eligible follow-up time (i.e. the time between the earliest date that the patient had six months of prior registration and the latest date that the patient had three months of follow-up). This was multiplied by a random number between zero and one and the resulting value was added to the start date of the eligible follow-up period. This date was used as the “index date” at which patient characteristics were measured.

### Cohort 2: exacerbation cases and controls

Within the study population, records of acute exacerbations were identified on the basis of Read codes (H312200: “Acute exacerbation of chronic obstructive airways disease”, H3y1.00: “Chron obstruct pulmonary dis wth acute exacerbation, unspec”) entered in the clinical and referral files. These records may have been entered prospectively or retrospectively (e.g. in the case of a patient presenting at an A&E unit with exacerbation). Individual exacerbation records were then grouped into episodes. Records within 28 days of a previous exacerbation record were considered to refer to the same episode. As such, the start of the episode was defined as the first record with no other exacerbation records in the previous 28 days and the end was defined as the last record with no other exacerbation records in the following 28 days. If a patient had several exacerbation episodes, one was randomly selected as the index event. Exacerbation events were excluded if they did not fulfil the inclusion criteria: (i) at least six months of prior registration in the primary care records, (ii) at least six months of prior coverage in HES, (iii) between 2005 and 2010, (iv) after COPD diagnosis, (v) patient was aged ≥40, (vi) prior to the end of primary care follow-up, (vii) prior to the end of HES coverage. Patients did not require any minimum amount of follow-up after the exacerbation.

Each exacerbation case was matched by age (within five years) and sex to up to three controls (patients not suffering exacerbation at the same time as the case) using incidence sampling. Three controls per case were selected to maximise statistical efficiency [13]. The index date of the controls was set to be that of the case (i.e. the start of the exacerbation episode). Controls with any record of exacerbation in primary care, or in HES in the primary diagnosis field, in the period six months prior to and three months post the index date of the case, were excluded. Each patient could only be a matched control for one case.

### Statistical analysis

Characteristics of both study cohorts were described using counts and percentages. Characteristics identified at the index date in the primary care data included age, sex, socio-economic status, BMI category (<20 underweight, 20–< 25 healthy weight, 25–< 30 overweight, 30+ obese), smoking status, prescriptions in the previous three months of any antibiotic, oral glucocorticoid therapy or anticholinergics (ipratropium bromide and tiotropium only), medical history of hypertension, diabetes, heart failure and depression, and most recent measure of forced expiratory volume in one second (FEV1 as classified by the Global initiative for Obstructive Lung Disease (GOLD) scale [14]).

For cohort 2, the characteristics listed above were compared between exacerbation cases and their matched controls using conditional logistic regression. A “missing value” category was assigned to categorical variables to enable the comparison of the proportion of missing data between exacerbation cases and their matched controls.

### Analysis of cohort 2: exacerbation cases

Resource utilisation on the exacerbation date, and in the week and three month period preceding it, was evaluated for the cases. This included prescribing of any antibiotic or oral corticosteroid, referrals to A&E units and community respiratory teams (as defined by Read codes and the NHS speciality recorded by the GP), hospitalisation with exacerbation as a primary diagnosis (as recorded in HES) with and without intubation or mechanical ventilation, lower respiratory tract infection, purulent sputum, increased sputum volume and increased dyspnoea.

Incidence rates of repeat exacerbation and all cause mortality in the three months following the exacerbation were calculated, stratified by the GOLD severity scale. The risk of repeat exacerbation and all mortality by GOLD severity was modelled using Cox regression, adjusting for age and gender. For both the incidence rates and the Cox regression follow-up time was censored at the end of data collection for each patient (i.e. the minimum of the date at which the patient left the practice and the last CPRD collection date from that practice).

The proportion of exacerbation cases prescribed an antibiotic on the index date was assessed by GP practice. The practice prescribing rate was plotted against the number of exacerbation cases, and confidence limits of the overall prescribing rate ±3 standard errors were superimposed. Prescribing variability was evaluated using a multilevel random intercept logistic model, with patients (level 1) clustered within practices (level 2), adjusting for patient characteristics known to be associated with antibiotic prescribing. The significance of the practice variation was assessed using a likelihood ratio test comparing the multilevel random intercept logistic model with a standard one level logistic regression model that did not take account of clustering within practice.

## Results

### Cohort 1: COPD population

The COPD cohort included 62,747 patients; half were aged ≥70 (52%) and half were male (Table 1). Half came from the two most deprived quintiles of the Index of Multiple Deprivation (51%), indicating relatively low socioeconomic status. The majority of patients were either current smokers (40%) or ex-smokers (49%) and half were overweight or obese (51%). High proportions of patients had been prescribed antibiotics (38%), anticholinergics (34%) or oral glucocorticoid therapies (17%) in the previous three months. Comorbidities were frequently recorded: 39% had a prior record of hypertension, 29% had a record of depression, 13% had a record of diabetes, and 8% had a record of heart failure. Just under half of patients were known to have severe (32%) or very severe COPD (13%).

**Table 1:** Characteristics of the COPD population, measured at the random index date

**Characteristic**	**COPD population**
	**62,747 (%)**
Age at random index date	
40–49	2,734 (4.4%)
50–59	9,253 (14.7%)
60–69	18,203 (29.0%)
70–79	19,615 (31.3%)
80+	12,942 (20.6%)
Male	33,241 (53.0%)
Quintile of Index of Multiple Deprivation	
0 (least deprived)	7,153 (11.4%)
1	11,199 (17.8%)
2	12,441 (19.8%)
3	14,801 (23.6%)
4 (most deprived)	17,033 (27.1%)
Body mass index at random index date	
Underweight	6,109 (9.7%)
Normal	18,556 (29.6%)
Overweight	18,640 (29.7%)
Obese	13,224 (21.1%)
Unknown	6,218 (9.9%)
Smoking status at random index date	
Non Smoker	6,586 (10.5%)
Ex Smoker	30,494 (48.6%)
Smoker	24,849 (39.6%)
Unknown	818 (1.3%)
Prescriptions in the previous three months	
Antibiotics	23,740 (37.8%)
Oral glucocorticoid therapies	10,645 (17.0%)
Anticholinergics	21,104 (33.6%)
Medical history	
Hypertension	24,245 (38.6%)
Diabetes	8,322 (13.3%)
Heart Failure	5,132 (8.2%)
Depression	18,282 (29.1%)
Forced expiratory volume in one second (FEV1) as proportion of predicted	
Mild COPD (≥80)	2,699 (4.3%)
Moderate COPD (50–<80%)	10,434 (16.6%)
Severe COPD (30–< 50%)	20,283 (32.3%)
Very severe COPD (<30%)	8,095 (12.9%)
Unknown	21,236 (33.8%)

### Cohort 2: exacerbation cases and controls

Within the study population, 12,609 patients were identified as having had an exacerbation episode; these cases were matched by age and gender to up to three controls each, resulting in 35,299 controls. Exacerbations were inversely associated with affluence (most affluent quintile compared to median quintile, matched odds ratio (OR) = 0.81 [95%CI 0.75–0.87]) and positively associated with being underweight (compared to healthy weight, OR = 1.13 [95%CI 1.06–1.21]) and current or past smoking (compared to non-smoking, OR = 1.42 [95%CI 1.30–1.55] and 1.38 [95%CI 1.27–1.50] respectively) (Table 2). Exacerbations were inversely associated with hypertension (OR 0.93 [95%CI 0.89–0.97]) and diabetes (OR 0.94 [95%CI 0.89–0.99]), and positively associated with depression (OR 1.17 [95%CI 12–1.22]). A higher proportion of cases had recently been prescribed oral glucocorticoid therapies (34%, OR 2.57 [95%CI 2.45–2.69]), anticholinergics (64%, OR 1.99 [1.91–2.08]) and antibiotics (51%, OR 1.95 [95%CI 1.87–2.03]) compared to the controls. In addition, 40% of cases were known to have severe or very severe COPD, compared to 27% of controls (very severe compared to mild COPD OR 2.12 [95%CI 1.95–2.32], severe compared to mild COPD OR 1.61 [95%CI 1.51–1.72]).

**Table 2:** Predictors of COPD exacerbations

**Characteristic**	**Exacerbation Cases**	**Controls**	**Unadjusted matched**
	**12,609 (%)**	**35,299 (%)**	**OR (95% CI)**
Age at exacerbation			
40–49	340 (2.7%)	985 (2.8%)	
50–59	1,473 (11.7%)	4,120 (11.7%)	
60–69	3,548 (28.1%)	9,831 (27.9%)	
70–79	4,399 (34.9%)	12,210 (34.6%)	
80+	2,849 (22.6%)	8,153 (23.1%)	
Male	6,240 (49.5%)	17,604 (49.9%)	
Quintile of Index of Multiple Deprivation			
0 (least deprived)	1,358 (10.8%)	4,564 (12.9%)	0.81 (0.75–0.87)
1	2,033 (16.1%)	5,964 (16.9%)	0.92 (0.86–0.99)
2	2,585 (20.5%)	7,022 (19.9%)	1.00
3	2,659 (21.1%)	7,479 (21.2%)	0.97 (0.91–1.03)
4 (most deprived)	3,974 (31.5%)	10,270 (29.1%)	1.05 (0.99–1.11)
Body mass index at exacerbation			
Underweight	1,562 (12.4%)	3,795 (10.8%)	1.13 (1.06–1.21)
Normal	3,846 (30.5%)	10,610 (30.1%)	1.00
Overweight	3,747 (29.7%)	10,747 (30.4%)	0.96 (0.91–1.01)
Obese	2,856 (22.7%)	8,295 (23.5%)	0.95 (0.90–1.01)
Unknown	598 (4.7%)	1,852 (5.2%)	0.89 (0.81–0.99)
Smoking status at exacerbation			
Non Smoker	840 (6.7%)	3,171 (9.0%)	1.00
Ex Smoker	7,141 (56.6%)	19,618 (55.6%)	1.38 (1.27–1.50)
Smoker	4,600 (36.5%)	12,414 (35.2%)	1.42 (1.30–1.55)
Unknown	28 (0.2%)	96 (0.3%)	1.12 (0.73–1.72)
Prescriptions in the previous three months			
Antibiotics	6,403 (50.8%)	12,193 (34.5%)	1.95 (1.87–2.03)
Oral glucocorticoid therapies	4,270 (33.9%)	5,871 (16.6%)	2.57 (2.45–2.69)
Anticholinergics	8,092 (64.2%)	16,738 (47.4%)	1.99 (1.91–2.08)
Medical history			
Hypertension	5,216 (41.4%)	15,163 (43.0%)	0.93 (0.89–0.97)
Diabetes	2,047 (16.2%)	6,028 (17.1%)	0.94 (0.89–0.99)
Heart Failure	1,314 (10.4%)	3,476 (9.8%)	1.07 (1.00–1.15)
Depression	4,624 (36.7%)	11,717 (33.2%)	1.17 (1.12–1.22)
Forced expiratory volume in one second (FEV1) as proportion of predicted			
Mild COPD (≥80)	1,832 (14.5%)	6,102 (17.3%)	1.00
Moderate COPD (50–<80%)	4,245 (33.7%)	13,838 (39.2%)	1.03 (0.96–1.09)
Severe COPD (30–<50%)	3,625 (28.7%)	7,533 (21.3%)	1.61 (1.51–1.72)
Very severe COPD (<30%)	1,362 (10.8%)	2,146 (6.1%)	2.12 (1.95–2.32)
Unknown	1,545 (12.3%)	5,680 (16.1%)	0.88 (0.82–0.96)

### Cohort 2: exacerbation cases

Table 3 shows the characteristics of exacerbation cases. A large number were prescribed an antibiotic (61%) or an oral corticosteroid (51%) on the date of the exacerbation record (70% of patients received either or both). The majority of exacerbations were managed in the primary care setting, as only 10% were referred to A&E, and only 7% were admitted to hospital for the COPD exacerbation, in the 7 days prior to and including the date of the exacerbation record.

**Table 3:** Characteristics of the COPD exacerbation cases

	**N (%)**
	**(total number of cases = 12,609)**
**Characteristic**	
Purulent sputum:	
On index date	171 (1.4%)
7 days prior to index - index date	230 (1.8%)
Lower respiratory infection:	
On index date	272 (2.2%)
7 days prior to index - index date	578 (4.6%)
90 days prior to index - index date	2,462 (19.5%)
Antibiotic prescribing:	
On index date	7,705 (61.1%)
7 days prior to index - index date	8,346 (66.2%)
90 days prior to index - index date	10,452 (82.9%)
90 days prior to index – 1 day before index	2,747 (21.8%)
Oral corticosteroid prescribing:	
On index date	6,444 (51.1%)
7 days prior to index - index date	6,869 (54.5%)
90 days prior to index - index date	8,511 (67.5%)
Referral to community respiratory team:	
7 days prior to index - index date	6 (<0.1%)
Referral to Accident and Emergency:	
On index date	888 (7.0%)
7 days prior to index - index date	1,239 (9.8%)
Hospitalisations for COPD exacerbation:	
7 days prior to index - index date	832 (6.6%)
With intubation or mechanical ventilation	33 (0.3%)

The incidence rates of repeat exacerbation and all cause mortality were 13.0 and 6.1 per 10,000 person-years, respectively (Table 4). After adjustment for age and sex, the risk of outcomes was seen to be highest amongst patients with very severe COPD.

**Table 4:** Outcomes (repeat COPD exacerbation and mortality) in the three months following COPD exacerbation by FEV1

**Outcome**	**FEV1 severity**	**Number of events (numerator)**	**Incidence rate per 10,000 person years**	**Age and sex adjusted hazard ratio**
Repeat COPD exacerbation	Mild	123	12.2 (10.2–14.5)	1.00
	Moderate	256	10.9 (9.6–12.3)	0.90 (0.72–1.11)
	Severe	287	14.6 (13.0–16.4)	1.22 (0.99–1.51)
	Very severe	129	17.8 (15.0–21.2)	1.51 (1.18–1.94)
	Unknown	103	12.4 (10.2–15.0)	1.01 (0.78–1.32)
All cause mortality	Mild	75	4.9 (3.9–6.1)	1.00
	Moderate	103	2.9 (2.4–3.5)	0.60 (0.45–0.81)
	Severe	192	6.4 (5.5–7.4)	1.26 (0.97–1.65)
	Very severe	107	9.6 (8.0–11.6)	2.14 (1.59–2.88)
	Unknown	166	13.2 (11.3–15.3)	2.23 (1.69–2.93)

**Figure 1:**
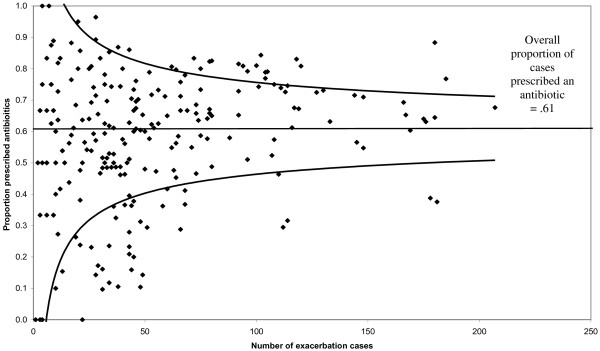
Variability of GP practice prescribing rates of antibiotics to COPD exacerbation cases with superimposed confidence limits (±3 standard errors) for all 237 practices with at least one case.

Whilst 61% of exacerbation cases were prescribed antibiotics, this proportion varied considerably between the 237 GP practices (interquartile range, 48–73%). In the 92 practices with at least 50 COPD patients, the proportion varied between 29% and 88% (interquartile range, 56–74%). Figure 1 shows a large proportion (25%) of GP practices with a prescribing rate outside that which might be expected, as indicated by their location outside of the confidence limits. GP practice explained 15% (95%CI, 12–19%) of the variability in antibiotic prescribing, after adjustment for patient characteristics (COPD hospitalisation, increased dyspnoea, age 80+, recent prescribing of oral glucocorticoid therapy or antibiotics, COPD severity, heart failure, underweight; all found to be inversely associated with antibiotic prescribing). The likelihood ratio test, comparing models with or without GP practice as a level, confirmed that clustering by GP practice was statistically significant (p < 0.001).

## Discussion

### Summary of main findings

This study of linked primary and secondary care databases provides an important perspective on the management and outcomes of COPD exacerbations. The results show that patients with an exacerbation recorded have higher recent prescribing rates (of oral glucocorticoid therapies, anticholinergics and antibiotics), and crucially, more severe COPD as measured by FEV1, which is shown to be associated with repeat exacerbation and all cause mortality. Whilst the majority of exacerbations are managed by GPs, there is significant variation between GP practices in the proportion of patients who receive a prescription for an antibiotic, which cannot be explained by patient characteristics alone.

### Strengths and limitations of the study

The large scale of the CPRD primary care database allowed analysis both at the patient and practice level. The representativeness and high quality of the database has been shown previously, indicating results are likely to be generalisable to England as a whole [9-11]. In addition, the linkage of primary care to secondary care data allowed for a broader analysis of resource utilisation, including whether patients received intubation or mechanical ventilation. However, some variables associated with antibiotic prescribing were not available, for example, nutritional status, certain biomarkers, and GP characteristics.

FEV1 was unknown for approximately 12% of exacerbation cases, and 34% of the COPD population overall. Whilst further FEV1 data may be recorded in the free text of the primary care data, which was not used in this study, prospective studies may wish to consider providing patients with spirometry devices to allow them to record their own measurements over time. However, in those patients with FEV1 measurements recorded by their GP, a clear relationship was seen between severity and poor outcomes, including exacerbations and mortality. This is in line with a recent review that found FEV1 a consistently strong predictor of exacerbation, hospitalisation and mortality amongst patients with COPD [15].

Despite purulent sputum being a commonly reported symptom of exacerbation, only a very small proportion of cases had this recorded by their GP [3]. Prospective studies should encourage GPs to record whether sputum is purulent or not, to enable a more robust analysis of the relationship with prescribing and outcomes.

This study was restricted to antibiotic prescriptions given by GPs, with the main analysis focusing on prescriptions on the exacerbation index date. This would not have captured prescriptions given on hospital discharge, provided in outpatient or home care settings, or those previously “stockpiled” by the patient.

### Comparison with existing literature

The significant variation between practices in antibiotic prescribing rates described in this study is consistent with the Audit Commission report of GP prescribing behaviours [16]. Prescribing rates are driven by a range of factors including physician’s knowledge, experience and uncertainty, time pressures, and varied approach to the role of patients in decision-making [17,18]. Conversely, a cohort study investigating the total number of prescriptions given to a patient by their GP found that most of the practice level variation could be explained by the patient’s age, sex and morbidity [19]. However, studies focusing specifically on antibiotic prescribing rates have demonstrated wide variation between practitioners that cannot be explained by differences in the epidemiology of infections, populations, or case mix, both in the UK and around the world [20-25].

## Conclusions

This study supports the need for a randomised trial to establish the role of antibiotics in treating mild or moderate COPD exacerbation, and ensure prescribing is evidence based rather than reliant on prescriber preference. A pragmatic trial using routinely collected EHR data appears feasible, as the COPD patients with exacerbation records were demographically representative of the COPD population overall, and disease severity recorded by the GP was strongly associated with both exacerbations and mortality.

## Competing interests

The study was funded by the NIHR HTA programme as part of implementing a pilot pragmatic randomised trial for the comparative effectiveness of antibiotics in patients with an exacerbation of chronic obstructive pulmonary disease and non-purulent sputum.

CPRD has received funding from the Medicines and Healthcare products Rregulatory Agency, Wellcome Trust, Medical Research Council, National Institute for Health Research Health Technology Assessment programme (NIHR HTA programme), Innovative Medicine Initiative, UK Department of Health, Technology Strategy Board, Seventh Framework Programme EU, various universities, contract research organisations and pharmaceutical companies. The department of Pharmacoepidemiology & Pharmacotherapy, Utrecht Institute for Pharmaceutical Sciences has received unrestricted funding for pharmacoepidemiological research from GlaxoSmithKline, Novo Nordisk, the private-public funded Top Institute Pharma (http://www.tipharma.nl, includes co-funding from universities, government, and industry), the Dutch Medicines Evaluation Board, and the Dutch Ministry of Health.

## Authors’ contributions

RB, SE and TPvS conceived, designed and analysed the study and drafted the manuscript. All authors contributed to the interpretation of results, and revising and approving the manuscript.

## Pre-publication history

The pre-publication history for this paper can be accessed here:

http://www.biomedcentral.com/1471-2466/13/32/prepub
